# Genome sequence of *Asaia bogorensis* strain SC1 isolated from an *Aedes aegypti* mosquito crop

**DOI:** 10.1128/MRA.00509-23

**Published:** 2023-10-16

**Authors:** Souvik Chakraborty, Joshua B. Benoit, Annette R. Rowe, Joshua D. Sackett

**Affiliations:** 1 Department of Biological Sciences, University of Cincinnati, Cincinnati, Ohio, USA; University of Maryland School of Medicine, Baltimore, Maryland, USA

**Keywords:** mosquito microbiome, crop, metabolic pathways, antibiotic resistance, genomic characterization

## Abstract

Understanding microbe-host interactions is key to combating disease transmission by mosquitoes. Here, we report the genome sequence of *Asaia bogorensis* strain SC1 isolated from a human-blood-fed *Aedes aegypti* mosquito crop. Metabolic pathway characteristics of aerobic respiration were present in the genome, along with multiple putative antibiotic resistance mechanisms.

## ANNOUNCEMENT

The dipteran crop, a food storage organ found in mosquitoes, harbors a diverse microbiome that can impact various aspects of host physiology and ecology (1 – 3). However, microbial metabolism in the crop remains poorly characterized, especially regarding its potential role in influencing mosquito-borne disease transmission (4). Here, we report the genome sequence of *Asaia bogorensis* strain SC1, isolated from a human-blood-fed *Aedes aegypti* mosquito crop. Importantly, *Asaia* sp. is dominant in the crop of *Ae. aegypti* (1), suggesting a critical role for these bacteria in the organ.

*Asaia bogorensis* strain SC1 was isolated from human-blood-fed (University of Cincinnati IRB 2021-0971) *Aedes aegypti* mosquito crop. Briefly, the crop was dissected from a mosquito using ethanol-flamed forceps, homogenized in 1× PBS pH = 7.4, and inoculated on Luria-Bertani (LB) agar at 30°C. To ensure a pure culture, individual colonies were sequentially streaked on fresh LB plates three times. DNA was extracted from overnight LB broth cultures (30°C, 200 RPM) using the Qiagen DNeasy PowerSoil Kit (Qiagen, Germantown, MD) and quantified via Qubit (ThermoFisher Scientific, Waltham, MA). For long-read sequencing, the Native Barcoding Kit 24 V14 Kit (Oxford Nanopore Technologies, Oxford, UK) was used to barcode samples and prepare libraries. DNA was not sheared or size selected prior to library prep. Sequencing was performed with an R10.4.1 flow cell (FLO-MIN114) and minION device under high-accuracy mode (280 bp/s). Basecalling was performed using Guppy 6.4.6, and reads with quality scores <7 were removed. Reads were filtered with Filtlong 0.2.1 (5) to remove reads <2,000 bp and the lowest-quality reads comprising 10% of all sequenced bases. Short-read sequencing libraries were prepared with Illumina DNA Prep Kit (San Diego, CA), barcoded with 10 bp UDI indices, and sequenced on an Illumina NovaSeq using 2 × 150 sequencing chemistry (SeqCenter, Pittsburgh, PA). Short-read sequence quality was visualized with FastQC 0.12.0 (6), and mean sequence quality was >Q30 at all positions, so no further quality filtering was conducted. Nanopore long-read sequences were assembled using Flye 2.9.1 (7). Illumina reads were aligned to the long-read assembly with Burrows-Wheeler Aligner 0.7.17 (8). Pilon 1.23 (9) was used to polish the assembly with parameter --fix all. In total, three rounds of polishing were conducted. Genome statistics and assembly quality were determined with QUAST 4.4 (10) and CheckM 1.0.18 (11). The genome was annotated with NCBI PGAP 6.4 (12). Potential metabolic pathways were identified using KEGG’s GhostKOALA tool (13). Taxonomy was assigned with GTDB-Tk 2.2.5 (14). Default parameters were used unless otherwise specified. Computing resources were supplied and maintained by the Ohio Supercomputer Center (15) and the DOE Systems Biology Knowledgebase (16).

The SC1 genome is 3.4 Mbp with 59.69% GC content (Table 1). GTDB identified the nearest neighbor as *Asaia bogorensis* (96.36% average nucleotide identity). GhostKOALA identified complete pathways for central carbon metabolism, including glycolysis and pyruvate oxidation, TCA cycle, and the pentose phosphate pathway. A complete oxidative phosphorylation pathway was annotated. Interestingly, pyruvate decarboxylase (*pdc*) was absent, but other putative fermentation genes, aldehyde dehydrogenases (*aldB*) and alcohol dehydrogenase (*adhA*), were present. GhostKOALA identified genes potentially conferring resistance to vancomycin, beta-lactams, and cationic antimicrobial peptides. The genome sequence of *Asaia bogorensis* strain SC1 from the mosquito crop provides a valuable resource for further investigating host-microbe interactions of public health interest.

**TABLE 1:** Sequencing statistics

Parameter	Value
Raw Illumina reads accession no.	SRR23872088
Raw Nanopore minION reads accession no.	SRR23872085
GenBank accession no. (genome assembly)	GCA_030263835.1
No. of raw paired-end Illumina reads	4,199,454
Illumina estimated genome coverage	168×
No. of raw Nanopore MinION reads (total length, base pairs)	1,215,752,376
Nanopore MinION read length N50 (bp)^*a*^	5,017
Nanopore MinION estimated genome coverage	303
Assembly size (bp)	3,404,989
G + C content (%)	59.69
Estimated genome completeness (%)^*a*^	99.75%
Estimated contamination (%)^*a*^	0
No. of contigs	4
Contig sizes (bp)	3,090,019 229,757 72,572 12,641
No. of protein-coding genes	3,025
No. of tRNA genes	58
No. of rRNA operons	5

^*a*^ Determined with CheckM 1.0.18 (9).

## Data Availability

This study is registered under BioProject PRJNA945045. The raw Illumina and Nanopore sequencing reads have been deposited in the Sequence Read Archive and are available under accession numbers SRR23872088 and SRR23872085, respectively. The complete genome sequence with annotations has been deposited in GenBank under accession number GCA_030263835.1.
